# The anterior thalamus provides a subcortical circuit supporting memory and spatial navigation

**DOI:** 10.3389/fnsys.2013.00045

**Published:** 2013-08-30

**Authors:** Maciej M. Jankowski, Kim C. Ronnqvist, Marian Tsanov, Seralynne D. Vann, Nicholas F. Wright, Jonathan T. Erichsen, John P. Aggleton, Shane M. O'Mara

**Affiliations:** ^1^Trinity College Institute of Neuroscience, Trinity College DublinDublin 2, Ireland; ^2^School of Psychology, Cardiff UniversityCardiff, UK; ^3^School of Optometry and Vision Sciences, Cardiff UniversityCardiff, UK

**Keywords:** anterior thalamus, memory, spatial navigation, theta rhythm, head direction cells

## Abstract

The anterior thalamic nuclei (ATN), a central component of Papez' circuit, are generally assumed to be key constituents of the neural circuits responsible for certain categories of learning and memory. Supporting evidence for this contention is that damage to either of two brain regions, the medial temporal lobe and the medial diencephalon, is most consistently associated with anterograde amnesia. Within these respective regions, the hippocampal formation and the ATN (anteromedial, anteroventral, and anterodorsal) are the particular structures of interest. The extensive direct and indirect hippocampal-anterior thalamic interconnections and the presence of theta-modulated cells in both sites further support the hypothesis that these structures constitute a neuronal network crucial for memory and cognition. The major tool in understanding how the brain processes information is the analysis of neuronal output at each hierarchical level along the pathway of signal propagation coupled with neuroanatomical studies. Here, we discuss the electrophysiological properties of cells in the ATN with an emphasis on their role in spatial navigation. In addition, we describe neuroanatomical and functional relationships between the ATN and hippocampal formation.

## Introduction

That the hippocampal formation is vital for memory is undeniable. For this reason, understanding hippocampal learning mechanisms remains one of the principal objectives in neuroscience. However, this problem must be addressed from a broad perspective, i.e., one that includes the many connections of the hippocampal formation, some of which are now known to be critical for hippocampal mnemonic functions. The medial diencephalon is extensively connected with the hippocampal formation, damage to this area being frequently associated with anterograde amnesia (Aggleton and Sahgal, 1993; Aggleton and Brown, 1999). Within the medial diencephalon, the anterior thalamic nuclei (ATN) are an important part of the neuronal systems involved in spatial navigation (Clark and Taube, 2012), complementing their role in mnemonic functions (Buzsáki and Moser, 2013). This review will summarize some recent data concerning the anatomical and physiological properties of the anterior thalamic neurons, their role in spatial navigation, and their relevance to pathophysiological conditions associated with the ATN.

## General anatomy of thalamus

**Figure 1:**
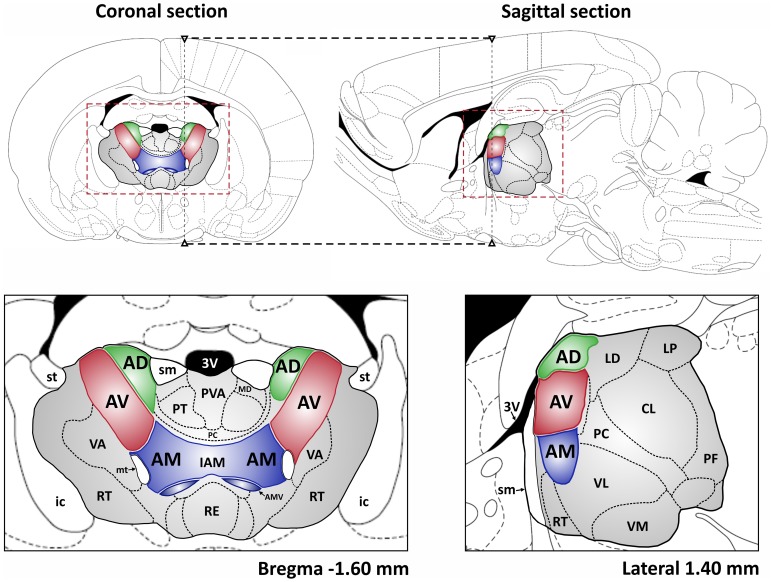
**The localization of the ATN in the rat brain. Top:** coronal and sagittal sections of rat brain are shown (Paxinos and Watson, 1998) with the ATN indicated in green, red and blue and whole area of the thalamus in gray. The dashed black lines depict the spatial relation between presented sections. The dashed red rectangles denote the extent of coronal and sagittal sections, respectively, presented below. Abbreviations: 3V, 3rd ventricle; AD, anterodorsal thalamic nucleus; AV, anteroventral thalamic nucleus; AM, anteromedial thalamic nucleus; AMV, anteromedial thalamic nucleus, ventral part; CL, centrolateral thalamic nucleus; IAM, interanteromedial thalamic nucleus; ic, internal capsule; LD, laterodorsal thalamic nucleus; LP, lateral posterior thalamic nucleus; MD, mediodorsal thalamic nucleus; mt, mammillothalamic tract; PC, paracentral thalamic nucleus; PF, parafascicular thalamic nucleus; PT, paratenial thalamic nucleus; PVA, paraventricular thalamic nucleus, anterior part; RE, reuniens thalamic nucleus; RT, reticular thalamic nucleus; sm, stria medullaris of the thalamus; st, stria terminalis; VA, ventral anterior thalamic nucleus; VL, ventrolateral thalamic nucleus; VM, ventromedial thalamic nucleus.

The thalamus is a bilateral, symmetrical structure comprising the majority of the diencephalon, with the medial thalamus being bordered, and in places split, by the third ventricle. The thalamus is classically divided into several groups of nuclei, described by their anatomical location: medial, lateral, ventral, and anterior, as well as the posterior (pulvinar) nuclei. This review focuses on the ATN, which is divided into the anterodorsal, anteroventral, and anteromedial nuclei, all located in the rostral part of the dorsomedial thalamus (Figure 1) (Morel et al., 1997; Wiegell et al., 2003). There is some uncertainty about the nuclei that comprise the ATN, with many authors regarding the lateral dorsal thalamic nucleus as part of the ATN due to its limbic associations (Morel et al., 1997). Some researchers have argued that the anteromedial nucleus is actually a part of the anteroventral nucleus (see Alelú-Paz and Giménez-Amaya, 2007), although in rodents and monkeys these two nuclei have clearly visible differences when examined in histological and immunochemical preparations. The three major nuclei within the ATN also have distinct patterns of connectivity.

## Histology

While the human anteroventral thalamic nucleus is distinguished by its homogenous and dense cell population (Morel et al., 1997), the neurons in anteromedial nucleus are larger and more widely dispersed. In contrast, the anterodorsal nucleus contains densely-packed small cells (Morel et al., 1997). All areas of the ATN show varying degrees of immunoreactivity to acetylcholinesterase, as well as the calcium-binding proteins calretinin, calbindin-D28K and parvalbumin (Morel et al., 1997; Fortin et al., 1998; Munkle et al., 2000; Alelú-Paz and Giménez-Amaya, 2007). In humans, the neuropil in ATN also stains variably in different areas for neuropeptides. These neuropeptide analyses reveal numerous substance P positive varicose fibers scattered throughout the ATN, in contrast to very few enkephalin positive varicose fibers (Alelú-Paz and Giménez-Amaya, 2007). Heterogeneity in morphological architecture and protein expression patterns within ATN may reflect regional differences in their functional organization with respect to the other thalamic nuclei and the cerebral cortex. Moreover, the varied morpho-chemical structure of the various ATN may underlie their different roles in the function of the limbic system.

## Connectivity to other structures

**Figure 2:**
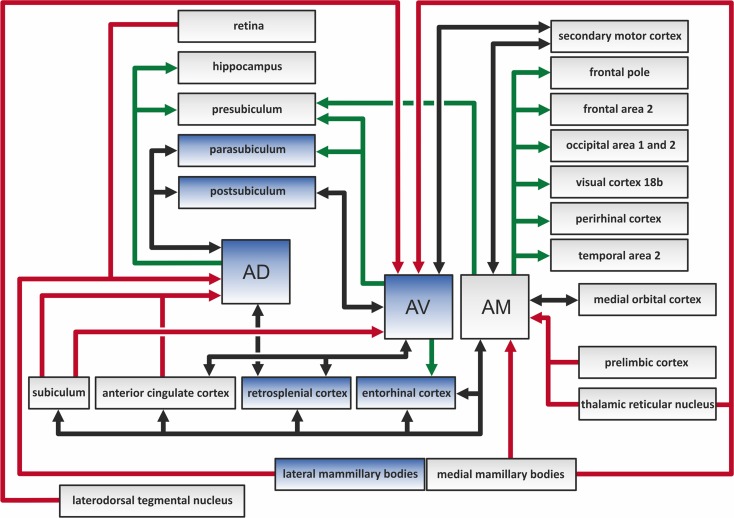
**The color-coded diagram presents the main direct connections of the anterodorsal (AD), anteroventral (AV), and anteromedial (AM) thalamic nuclei in the rat brain.** Black arrows depict reciprocal connections, green efferents, and red afferents of the three anterior thalamic nuclei (ATN). Structures in blue contain head direction cells, and so constitute a part of the hierarchically organized head direction system (Clark and Taube, 2012). The various indirect connections of the ATN, along with the connections between other highlighted structures, are not included in this scheme.

The ATN sits in the middle of a complex array of cortical and subcortical connections (Figure 2). Examples include the widespread links with frontal cortical areas, much of the cingulate cortex, and the hippocampal formation (Amaral and Cowan, 1980; Hicks and Huerta, 1991; Van Groen and Wyss, 1995). Many of these connections are reciprocal (Shibata and Naito, 2005). Especially dense inputs to the ATN arise from the retrosplenial cortex, the subiculum, and the mammillary bodies (Wright et al., 2010); the latter reach the thalamus via the mammillothalamic tract. The mammillary body inputs are particularly notable as it appears that almost every neuron within the structure projects to the ATN (Hopkins, 2005; Vann et al., 2007; Aggleton et al., 2010). However, the various projections to the ATN are often topographically specific (Wright et al., 2013). Previous rodent and primate studies had indicated that separate cell groups in the subiculum project to either the mammillary bodies or the anterior thalamus (Naber and Witter, 1998; Ishizuka, 2001; Aggleton et al., 2005). Wright et al. (2010) investigated this specificity and found distinct bands of projection to each area, i.e., the inputs are segregated. This same pattern of segregation extends to the inputs to the anteroventral and anteromedial nuclei, which often arise from the same structure but rarely from the same cells (Wright et al., 2013). The finding that the direct hippocampal projections to the mammillary bodies and ATN rely on the fornix (Aggleton et al., 2005, 2010; Saunders et al., 2005) is important as it has a direct bearing on how the impact of fornix damage upon cognition is interpreted (Tsivilis et al., 2008). A brief summary of some of the connections involving the different nuclei in the rodent anterior thalamus is summarized as follows (see also Figure 2):

### Anteromedial

Anteromedial nucleus receives projections from:

- medial mammillary bodies (Watanabe and Kawana, 1980; Seki and Zyo, 1984)
- rostral dorsal reticular nucleus (Shibata, 1992)
- prelimbic and medial orbital cortices (Shibata and Naito, 2005)
- anterior cingulate and dysgranular retrosplenial cortex (Shibata, 1993b; Shibata and Naito, 2005; Wright et al., 2010)
- secondary motor cortices (Shibata and Naito, 2005)
- entorhinal cortex (Wright et al., 2010)
- subiculum (Wright et al., 2010)

Anteromedial nucleus projects to:

- frontal area 2 (Shibata and Kato, 1993), frontal polar and medial orbital cortex (Van Groen et al., 1999)
- anterior cingulate (Shibata and Kato, 1993) and dysgranular retrosplenial cortex (Shibata and Kato, 1993; Van Groen et al., 1999)
- entorhinal cortex (Shibata, 1993a; Shibata and Kato, 1993; Van Groen et al., 1999);
- perirhinal cortex (Shibata, 1993a; Van Groen et al., 1999)
- presubiculum, subiculum (Shibata, 1993a; Van Groen et al., 1999)
- visual cortex area 18 b (Van Groen et al., 1999)
- temporal area 2, occipital area 1 and 2 (Shibata, 1993a)
- (medial) secondary motor cortices (Shibata and Naito, 2005)

### Anterodorsal

Anterodorsal nucleus receives projections from:

- lateral mammillary bodies (Watanabe and Kawana, 1980; Shibata, 1992)
- subiculum, para-and postsubiculum (Seki and Zyo, 1984; Van Groen and Wyss, 1990a,c; Wright et al., 2010)
- retina (Conrad and Stumpf, 1975; Itaya et al., 1981, 1986)
- anterior cingulate cortex (Shibata and Naito, 2005)
- granular retrosplenial cortex (Wright et al., 2010)
- caudal dorsal reticular nucleus (Shibata, 1992)

Anterodorsal nucleus projects to:

- pre-, para-, and postsubiculum (Van Groen and Wyss, 1990a,c, 1995)
- hippocampus (Wyss et al., 1979; Amaral and Cowan, 1980)
- granular retrosplenial cortex (Van Groen and Wyss, 1990b; Shibata, 1993b; Van Groen and Wyss, 2003)

### Anteroventral

Anteroventral nucleus receives projections from:

- medial mammillary bodies (Watanabe and Kawana, 1980)
- caudal dorsal reticular nucleus and laterodorsal tegmental nucleus (Shibata, 1992)
- subiculum and postsubiculum (Van Groen and Wyss, 1990c; Wright et al., 2010)
- anterior cingulate cortex, granular and dysgranular retrosplenial cortex (Van Groen and Wyss, 1990b, 2003; Shibata and Naito, 2005; Wright et al., 2010)
- secondary motor cortex (Shibata and Naito, 2005)

Anteroventral nucleus projects to:

- pre-, para-, and postsubiculum (Van Groen and Wyss, 1990c; Shibata, 1993a; Van Groen and Wyss, 1995)
- entorhinal cortex (Shibata, 1993a)
- anterior cingulate, granular and dysgranular retrosplenial cortex (Shibata, 1993b; Van Groen and Wyss, 2003)
- secondary motor cortex (Shibata and Naito, 2005)

## Functional considerations

**Figure 3:**
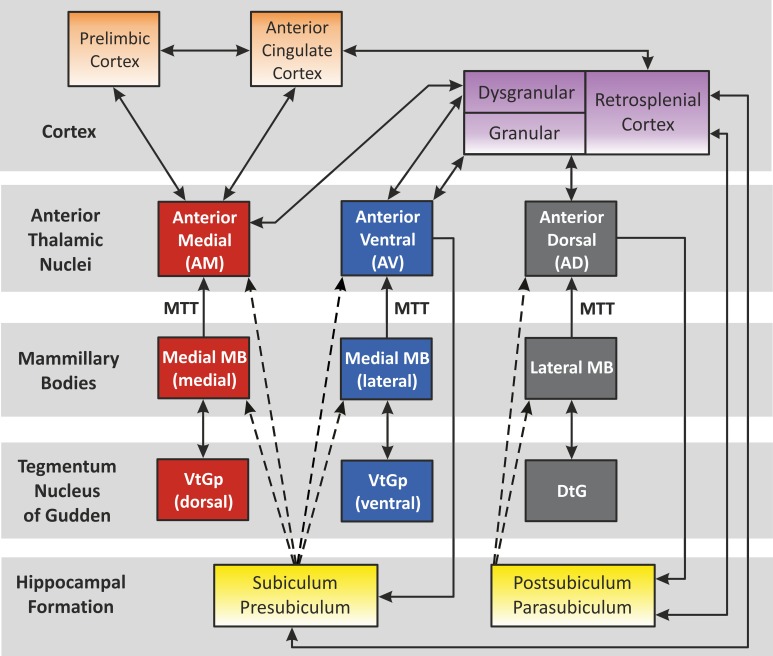
**The “extended-hippocampal system” proposed by Aggleton et al. (2010).** Color-coded diagram depicts how, in the rat, the hippocampal formation is associated with three sets of parallel mammillary body—anterior thalamic connections. Connectivity studies in the monkey brain (macaque) support the same overall scheme for primates (e.g., Vann et al., 2007). The connections solely conveyed in the fornix are shown as dashed lines. Double-headed arrows depict reciprocal connections. Abbreviations: DtG, dorsal tegmental nucleus of Gudden; MTT, mammillothalamic tract; VtGp, ventral tegmental nucleus of Gudden, pars posterior. (Note, the lateral dorsal thalamic nucleus has not been included above as, unlike the anterior thalamic nuclei, it receives few, if any, mammillary body inputs. The interoanteromedial nucleus has not been included given its uncertain status in the primate brain).

The circuit outlined by Papez (1937) is still highly relevant when considering the functional and cognitive aspects of systems involving the anterior thalamus. This circuit highlighted the following projections: hippocampal formation > mammillary bodies > anterior thalamus > cingulate cortex > parahippocampal gyrus > hippocampal formation. Since then, the anatomical definition of Papez' circuit has been further refined (Shah et al., 2012). The ATN still occupy an important position as established by studies using traditional fiber dissection techniques (Shah et al., 2012), as well as *in vivo* diffusion spectrum imaging (Granziera et al., 2011) of Papez' circuit. Although Papez originally suggested that this pathway underlay emotional processing by the brain, our current understanding of Papez' circuit suggests that it has a particular and special role in supporting the neural substrates of explicit learning and memory (Vertes et al., 2001; Shah et al., 2012). Aggleton and Brown (1999) developed the idea of an extended hippocampal-diencephalic network for the integration of information, with the ATN at its core. Subsequent models have proposed that the individual ATN can be functionally divided, forming a series of three parallel sub-systems (Aggleton et al., 2010) (Figure 3): (1) The anteromedial nucleus is predicted to form part of a largely feed-forward system that conveys integrated information from the hippocampal-diencephalic network to prefrontal areas, thereby taking part in higher cognitive and executive functioning; (2) The anteroventral system largely comprises a return-loop, with the main purpose being to perpetuate rhythmic theta activity to the hippocampal formation; (3) The anterodorsal nucleus is considered to encompass the head direction system. This description arises because cells in this nucleus exhibit electrophysiological compass-like properties, so that they display tuning to specific head directions, but not to location (Taube, 2007; Clark and Taube, 2012). The proposal is that the combined properties aid both spatial and mental navigation, with a different emphasis in different species (Aggleton et al., 2010).

## Spatial navigation role of anterior thalamic neurons—a critical part of the head direction system

Investigations into the roles of anterior thalamic neurons in spatial navigation were triggered by the discovery of cells in the postsubiculum that discharge as a function of the animal's head direction in the horizontal plane, but independent of its behavior and location in the environment (Ranck, 1984; Taube et al., 1990). Knowing that the postsubiculum contains reciprocal connections with the ATN (anterodorsal nucleus in particular) led to the suspicion that the anterior thalamus might also possess head direction cells. In 1995, Taube reported that such cells, referred to as head direction cells (because they only discharge whenever the animal points its head in a particular direction), were indeed present in the ATN (Taube, 1995). Head direction cells are believed to encode primary information for spatial orientation in the environment, namely an animal's perceived directional heading with respect to its environment (for review see Taube, 2007; Clark and Taube, 2012). So far, the largest proportions of head direction cells in the thalamus have been found in the anterodorsal and lateral dorsal thalamic nuclei, with additional head direction cells in the anteroventral nucleus (Taube, 2007; Tsanov et al., 2011a; Clark and Taube, 2012). Moreover, head direction cells are also found in cortical structures such as the postsubiculum, parasubiculum, retrosplenial, and medial entorhinal cortex, as well as in subcortical brain regions like the lateral mammillary nucleus (LMN) and dorsal tegmental nucleus of Gudden (DTG) (Clark and Taube, 2012).

There is now considerable evidence that the ATN are part of an interconnected circuit, which is organized hierarchically and is responsible for the propagation of head directional signals in the central nervous system (Taube, 2007; Clark and Taube, 2012). Such a notion is supported by experiments in which lesions of the lower structures of this circuitry (e.g., anterodorsal thalamus) completely abolished head direction cell activity in higher components (e.g., postsubiculum, parasubiculum or superficial layers of medial entorhinal cortex), whereas destruction of postsubiculum did not disrupt head direction signals in subcortical structures (Goodridge and Taube, 1997; Clark and Taube, 2011, 2012). Moreover, damage to the postsubiculum or retrosplenial cortex disrupted anterodorsal nucleus head direction cell tuning to visual landmark cues, suggesting that these cortical structures are important for the visual regulation of head direction cells activity in the anterior thalamus (Goodridge and Taube, 1997; Clark et al., 2010; Yoder et al., 2011b). The medial entorhinal cortex seems to be at the top of this hierarchical head direction system because, after lesion, the discharge characteristics of anterodorsal head direction cells were only mildly affected. Furthermore, entorhinal cortex lesions did not cause clear deficits in landmark processing or angular path integration (neural integration of head angular velocity signals) by anterodorsal head direction cells (Clark and Taube, 2011). Further evidence for the hierarchical organization of head direction cell circuitry comes from experiments in which lesions in “lower” structures, e.g., bilateral damage of the dorsal tegmental nucleus of Gudden or the lateral mammillary nucleus, abolished head direction cell activity in the anterodorsal thalamic nucleus (Blair et al., 1998, 1999; Bassett et al., 2007). In contrast to this general pattern of hierarchical organization, lesions in lateral dorsal thalamic nucleus had little effect on the firing properties of head direction cells in postsubiculum (Golob et al., 1998), whereas an intact anterodorsal thalamic nucleus is necessary for the presence of head direction cell activity in the postsubiculum (Goodridge and Taube, 1997).

Thalamic head direction cells are influenced by both external and internal sources of information (Taube, 2007; Yoder et al., 2011a). Although external cues exert strong influences on anterior thalamic head direction cells, these cells can maintain directional firing preferences in the dark and in new environments (Taube and Burton, 1995; Goodridge et al., 1998). This observation suggests that head direction cells are strongly influenced by internal sources of information, i.e., vestibular, proprioreception, or motor efference. One implication is that the vestibular system may be particularly important for this aspect of spatial navigation (Potegal, 1982). This hypothesis was verified experimentally by (Stackman and Taube, 1997), who recorded from head direction cells in the anterodorsal thalamus before and after neurotoxic lesions that destroyed the hair cells in the vestibular labyrinth. As a result, head direction cells in the anterior thalamus lost their directional specificity. Moreover, in lesioned animals, a new subset of neurons, characterized by intermittent firing bursts without specified directionality, was observed. The appearance of a new subset of cells in lesioned animals that were not recorded in intact animals suggests that head direction cells may alter their physiology in the absence of indirect vestibular input, and that other sensory systems (e.g., visual, somatosensory/tactile or olfactory) are unable to compensate for the loss of vestibular information in order to retain direction. The absence of head direction cell activity in animals with vestibular lesions persisted for up to 3 months post-surgery, indicating that indirect vestibular inputs remain crucial for anterior thalamic head direction cell function (Clark and Taube, 2012). However, in the anterodorsal thalamic nucleus of transgenic otoconia deficient *tilted* mice, which exhibit an impaired sense of linear acceleration and head tilt, directionally tuned cells were recorded (Yoder and Taube, 2009). Nevertheless, the head direction cells recorded in *tilt* mice often appeared to be unstable. These cells retained directional information for the duration of a single recording session, but often lost directionality across subsequent recording sessions. These experiments (Yoder and Taube, 2009) provided the first conclusive evidence that the otolith organs are important for maintenance of a robust head direction signal.

One of the main questions that emerged after the discovery that the anterodorsal head direction signal is dependent on indirect vestibular inputs was: Are anterodorsal head direction cells activated in the same manner during active and passive movement? Initially, (Knierim et al., 1995) and Taube (1995) both reported substantial reductions in firing rates during passive rotation, producing near or complete suppression of the anterodorsal head direction response when the animal's body was tightly restrained except its head. Reductions in firing rates during passive rotation were also observed in the postsubiculum and retrosplenial cortex (Chen et al., 1994; Golob et al., 1998). In contrast to these observations, (Zugaro et al., 2001) found only mild inhibition of anterodorsal head direction cell firing, with peak firing rates reduced by only 27% and no loss of directional responding during unrestrained passive movement. (Bassett et al., 2005) found only a 23% reduction in the peak firing rates of anterodorsal head direction cells when the animals were passively moved while loosely restrained. The above observations suggest that the tight restraint of the animal may, in itself, be a factor which decreases firing rate of anterodorsal head direction cells. However, in the studies by Knierim et al. (1995) and Taube (1995), the head of the animal was not fully immobilized while the trunk was tightly restrained. Therefore, Shinder and Taube (2011) prepared a rotatable, horizontal plane platform that was equipped with an immobilizing tube for the trunk and holder for the head. Before the recording session, the rat was immobilized and its head fixed to the platform by a bar connected to the restraint bolt, which had been previously mounted to the skull. Experiments revealed that passive movement during head-fixed restraint did not reduce anterodorsal head direction cell firing, relative to active movement (Shinder and Taube, 2011). Moreover, anterodorsal head direction cell responses were also maintained during passive movement in the dark, suggesting that visual, motor, and proprioceptive inputs are not necessary to generate direction-specific responses in head direction cells. This experiment further supports the hypothesis that indirect vestibular input is crucial for head direction cell activity in the anterodorsal thalamus.

Another cell type relevant for spatial navigation is the “place cell.” Place cells discharge when an animal is in a particular location in the environment (O'Keefe and Dostrovsky, 1971). So far, “true” place cells have only been recorded in the hippocampus (Clark and Taube, 2012). However, the anterior thalamus, as part of the limbic system and head direction system, may contribute to the function of hippocampal place cells. Several theories have suggested that place cells use the signal from the head direction system to establish and maintain place-field activity (McNaughton et al., 1996; Touretzky and Redish, 1996; Sharp, 1999). Calton et al. (2003) reported that, after lesions of the anterodorsal thalamic nucleus, place cells continued to exhibit location specific activity, but the place fields were somewhat degraded and cells were more directionally-sensitive. These observations suggest that input from anterodorsal head direction cells may be important for processing and integrating spatial information within the hippocampal circuits containing place cells.

## Theta rhythm in the anterior thalamic nuclei

The nuclei within Papez' pathway that mediate head direction signals are closely paralleled by those adjacent nuclei mediating theta rhythm (a sinusoidal oscillation of 6–12 Hz). Theta rhythm is considered to play a critical role in spatial and non-spatial mnemonic functions of the limbic system (Burgess et al., 2002; Buzsaki, 2005). Both circuits (HD vs. theta) include the tegmental nuclei of Gudden (dorsal vs. ventral), the mammillary bodies (lateral vs. medial), the ATN (anterodorsal vs. anteroventral) and the subicular/entorhinal cortices (Swanson and Cowan, 1977; Witter et al., 1990; Shibata, 1993b; Van Groen and Wyss, 1995; Gonzalo-Ruiz et al., 1997; Van Groen et al., 1999). Electrophysiological studies in rats support this idea, because plasticity between sequentially-activated hippocampal place cells occurs during theta epochs (Mehta et al., 2000; Ekstrom et al., 2001), implicating the theta cycle as an information quantum (Skaggs et al., 1996; Buzsaki, 2002). Theta rhythm commonly modulates the spike trains of spatially-tuned neurons such as hippocampal place cells (O'Keefe and Dostrovsky, 1971), entorhinal grid cells (Hafting et al., 2005), and border cells (Savelli et al., 2008; Solstad et al., 2008). These neurons, together with HD cells, are believed to participate in computing the animal's location in the environment by integrating its movement velocity over time, the process referred to as path integration (McNaughton et al., 1996; Etienne and Jeffery, 2004).

**Figure 4:**
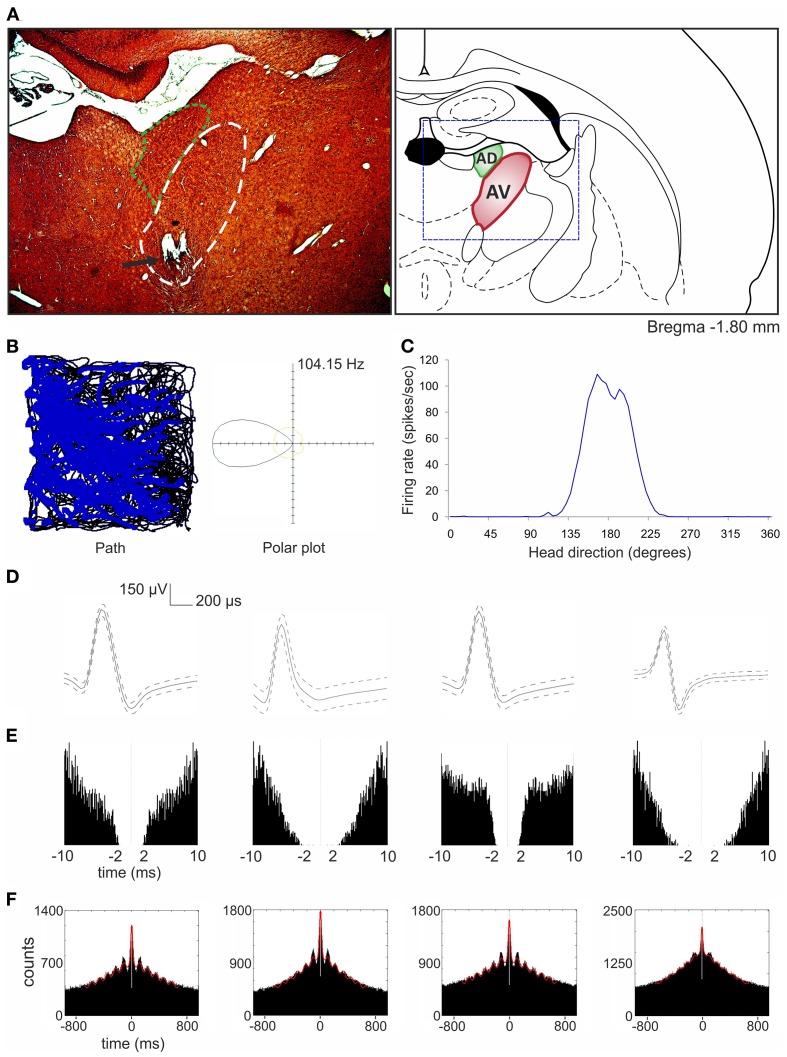
**Head direction-by-theta cells recorded in anteroventral thalamic nucleus (Tsanov et al., 2011a). (A)** Anatomical location of chronically implanted tetrodes aimed at anteroventral nucleus (bundle of eight tetrodes). On the left, the histological slide showing the location of chronically implanted tetrodes marked with the black arrow. Area of anteroventral nucleus is indicated with white dashed line and anterodorsal nucleus with green dashed line. On the right, location of anteroventral and anterodorsal nuclei is shown on the modified section from the rat brain atlas (Paxinos and Watson, 1998). The dashed blue rectangle denotes the extent of the histological section on left. **(B)** On the left, the path of the animal (black line) with superimposed firing activity of head direction-by-theta unit (blue dots) recorded during 16-min session in a square arena (64 × 64 × 25 cm). On the right, the polar plot represents the distribution of time heading in different directions across all time bins of the trial (yellow) and the distribution of head directions for time bins when a spike was recorded from the cell (black). **(C)** The same signal can be plotted as firing rate vs. head direction tuning plot for head direction-by-theta units. **(D,E)** The spike waveform **(D)** and the autocorrelogram of spiking activity calculated for 10/10 ms **(E)** for four anteroventral head direction-by-theta units, respectively. For the spike waveform, the solid curve represents the mean, and the dashed curve represents the SD. The clear isolation of the neuronal extracellular response was identified by the absence of correlations within the first 2 ms of the refractory period. **(F)** The 1000 ms autocorrelograms of four head direction-by-theta units. The fitted vertical red line indicates the relative amplitude of the sinusoid component of the autocorrelogram, visualizing the degree of autocorrelogram rhythmicity.

So far, the anterodorsal thalamic nucleus is the best-described thalamic nucleus with respect to the electrophysiological properties of its neurons in freely moving animals. A particular focus on this nucleus stems from the fact that it contains high numbers of head direction cells (Taube, 2007; Clark and Taube, 2012). Single-unit recordings in other ATN (anteroventral and anteromedial) in urethane-anesthetized rats reveal that some anteroventral neurons tend to fire in theta-rhythmic manner (Vertes et al., 2001). This observation was confirmed by single-unit recordings both in freely moving rats foraging for food pellets and during naturally occurring sleep (Tsanov et al., 2011b). An identified subgroup of anteroventral neurons was strongly entrained by theta oscillations and synchronized their bursting activity in theta range. Moreover, theta and spindle oscillations differed in their spatial distribution within the anteroventral nucleus, suggesting that separate cellular sources are responsible for these oscillations. Approximately 23% of anteroventral neurons were assigned to the slow- and fast-spiking bursting units that are selectively entrained to theta rhythm (Tsanov et al., 2011b). Importantly, Tsanov et al. (2011a) also reported large subpopulation of head direction cells (39%) in the anteroventral thalamic nucleus that exhibit rhythmic spiking in the theta range. This class of units is termed head direction-by-theta cells, which discharge predominantly in spike trains at theta frequency whenever the animal is heading/facing in the preferred direction (Figure 4). Neurons possessing both theta and head-directional properties have been described earlier at the higher level of this circuitry, namely the presubicular/parasubicular region (Cacucci et al., 2004; Boccara et al., 2010). Tsanov et al. (2011a) showed for the first time that the integration of head-directional and theta information takes place at the level of the anteroventral thalamic nucleus. It is likely that this integrated information is sent in an ascending projection within Papez' circuit and so contributes to the complex firing properties of the presubiculum and parasubiculum as well as other parts of the extended hippocampal formation. Moreover, it is possible that non-directional theta cells from anteroventral thalamic nucleus may contribute to the priming of retrosplenial cells, thus magnifying the influence of anterodorsal head direction cells on neurons in retrosplenial cortex (Albo et al., 2003). Directional information may also be particularly important for animals engaged in locomotor/exploratory behaviors (theta states) and less during non-locomotor activities (non-theta states). This notion is supported by work of Zugaro et al. (2001), who reported that anterodorsal head direction cells fire at significantly higher rates during active, compared to passive, motion of rats. However, Shinder and Taube (2011), using their platform for full immobilization of the rat, found that the firing of anterodorsal head direction cells does not differ between active and passive movement. The contradictions concerning the activity of HD cells in active vs. passive movement in fully vs. partially restrained animals still needs to be clarified. Available data suggest that HD cells from anterodorsal thalamic nucleus respond solely to the perceived head direction. However, the available data does not allow one to fully exclude the influence of other factors on HD cells activity such as theta oscillations or input information from other structures that may appear during active movement. Clearly, the role of theta oscillations in the ATN on the function of head direction cells at all levels of the hierarchical head direction circuitry remains to be fully elucidated.

**Figure 5:**
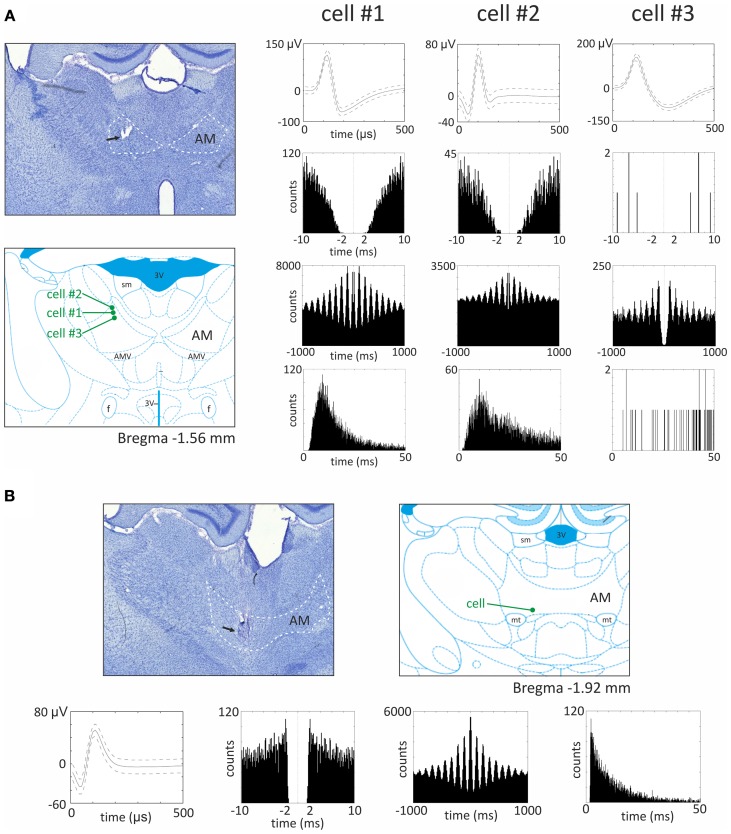
**Theta modulated cells from the anteromedial thalamic nucleus. (A)** Recording sites and three examples of theta modulated cells recorded in the superficial part of anteromedial nucleus. On the left, the histological slide showing the location of chronically implanted tetrodes marked with the black arrow. In this case, three theta modulated cells were recorded in the superficial part of anteromedial nucleus. Estimated location of recorded cells is marked below by green dots on the section from rat brain atlas (Paxinos and Watson, 2006). On the right, parameters of three theta modulated cells recorded in this rat are presented. From the top for each cell, the waveform, autocorrelation for 10 ms, autocorrelation for 1000 ms and interspike interval histogram (ISIH) are presented. All three cells exhibit different firing rate and waveforms, but all are modulated in the theta rhythm frequency, which is visible as 6–10 peaks on 1000 ms autocorrelogram. The ISIH indicates that recorded cells were not bursting neurons (there is no peak of firing before 5 ms). **(B)** Recording site and example of theta modulated cell recorded in the bottom part of anteromedial nucleus. In the top, the histological slide shows the location of chronically implanted tetrodes, marked with the black arrow. In this case, a theta modulated cell was recorded in the bottom part of anteromedial nucleus (see also estimated position of the cell on the right). Below, the waveform, autocorrelation for 10 ms, autocorrelation for 1000 ms, and ISIH are presented. The 1000 ms autocorrelogram indicates that this cell was modulated in the frequency of theta rhythm and ISIH clearly shows that this cell is a bursting neuron. Recordings were performed in rats chronically implanted with driveable 32-channel microelectrodes organized in tetrodes. Each recording session lasted 20 min and was performed in freely moving rats foraging for food pellets in a circular arena (96 cm diameter). Abbreviations: AM, anteromedial thalamic nucleus; 3 V, third ventricle; AMV, anteromedial thalamic nucleus, ventral part; f, fornix; mt, mammillothalamic tract; sm, stria medullaris of the thalamus.

Vertes et al. (2001) initially reported that, in urethane-anesthetized rats, it was not possible to record theta modulated cells in the anteromedial and anterodorsal thalamic nuclei, as opposed to the anteroventral nucleus. However, in later experiments also performed in urethane-anesthetized rats, Albo et al. (2003) found theta-modulated cells in the anteromedial and anterodorsal thalamic nuclei. Our unpublished observations from recordings in freely-moving rats implanted with driveable microelectrodes confirm the presence of theta modulated cells in the anteromedial thalamic nucleus (see Figure 5 for examples of recorded units). Moreover, in the ATN, (Welday et al., 2011) recorded theta-modulated cells with theta cell burst frequencies that varied as the cosine of the rat's movement direction, and this directional tuning was influenced by landmark cues.

## Neuropathological considerations

The importance of the ATN for memory was shown by Harding et al. (2000), who studied the post-mortem brains of Korsakoff's psychosis patients. This condition, which is typically seen in alcoholics, causes an amnesic syndrome characterized by persistent anterograde episodic memory loss, but with a relative preservation of semantic memory, intelligence, and procedural behavior. Harding et al. found that ATN atrophy was consistent with the amnesia in Korsakoff's patients, but was not found in other, closely-related alcoholic conditions (e.g., Wernicke's encephalopathy) that do not produce a persistent amnesia. Anterograde memory impairments (e.g., in delayed recall) were reported in twelve patients with infarcts involving the ATN (Ghika-Schmid and Bogousslavsky, 2000), while a review of thalamic stroke patients confirmed that damage involving the mammillothalamic tract was the best predictor of amnesia (Carlesimo et al., 2011).

Both lesion and stimulation studies have played a vital role in accruing knowledge about the function of certain brain structures. With regards to the ATN, the importance of these nuclei for spatial functioning and memory has been demonstrated in many experiments over the last two decades. One of the primary arguments for the functional significance of the hippocampus-diencephalic linkage is found in rodent studies, where discrete lesions in the hippocampus, mammillary bodies, fornix, and ATN all disrupt performance on spatial learning tests such as alternation, but with varied severity (Aggleton and Sahgal, 1993; Aggleton et al., 1995, 2010; Byatt and Dalrymple-Alford, 1996; Sziklas and Petrides, 1998; Vann and Aggleton, 2003). Deep-brain stimulation (DBS) of the ATN can also disturb spatial alternation performance by rats (Hamani et al., 2010). Moreover, lesions in the hippocampus, fornix, and ATN disrupt performance on tests of temporal order discrimination (Fortin et al., 2002; Charles et al., 2004; Wolff et al., 2006; Aggleton et al., 2010).

The functional importance of the ATN in some frequent neuropathological problems has been shown by applying DBS to these nuclei in epilepsy patients, a procedure of particular relevance for those who are not eligible for respective surgery (Hodaie et al., 2002). The world-wide prevalence of epilepsy is approximately 1% and approximately 30% of patients do not respond to current pharmaceutical interventions (Kwan and Brodie, 2000). Further clinical studies have shown significant reductions in event frequency (Lee et al., 2012) after DBS of ATN. Although the clinical study by Lee et al. also tested for effects on seizure types and for anticonvulsant actions, the low number of participants resulted in no significant results for these categories. The exact mechanism of the clinical benefit of DBS to the ATN is unclear, but it is more likely to concern a larger network effect involving several brain regions, rather than being simply a local effect within the ATN and hippocampal-diencephalic system. Evidence for this can be taken from the change in motor excitability seen in epileptic patients who received bilateral DBS in the ATN, while their TMS-evoked motor potentials were recorded (Molnar et al., 2006).

In the pilocarpine epilepsy rodent model, stimulation of the ATN reduced seizure activity (Fisher et al., 2010; Jou et al., 2013) and protected against status epilepticus (Hamani et al., 2004). In another rodent epilepsy model, where seizures were induced by electrical stimulation of the basolateral amygdala, low-frequency bilateral ATN stimulation significantly reduced the severity and incidence of seizures (Zhong et al., 2011). Application of bilateral high-frequency stimulation in rats to the ATN after amygdala-induced seizures (e.g., replicating clinical post treatment application) decreased the incidence and duration of subsequent seizures (Zhang et al., 2012a). Another study by the same group showed that unilateral high frequency stimulation of the ATN before amygdala-induced seizures inhibited the induced seizures, and was concluded to suppress susceptibility to seizures (Zhang et al., 2012b). However, Lado (2006) reported that the effects of DBS in acute chemoconvulsant model of seizures in rodent may differ from chronic epilepsy conditions. Lado used kainate-induced chronic seizures in rats and tested the effects of bilateral anterior thalamic DBS. In contrast to previously reported benefits, Lado (2006) showed a 2.5 times increase in seizure frequency, compared to their chronic baseline after DBS in the ATN. The author highlighted this difference in their results as important with regards to both the location of the epileptic focus, phenotype, neuronal injuries present, and the difference between species. Since then, several clinical studies have shown the benefits of applying DBS in the ATN in epileptic patients; the recent SANTE trial review (Stimulation of ATN for Epilepsy) concluded that bilateral stimulation of the ATN reduced seizures on average by more than 50% through two years of this study (Fisher et al., 2010).

## Summary

The ATN form a pivotal part of Papez' circuit, with widespread limbic connections forming an “extended hippocampal formation.” Based on existing anatomical and electrophysiological data, we suggest there are, at least, three parallel hippocampal—anterior thalamic circuits (Aggleton et al., 2010). Studies of diencephalic amnesia reinforce the crucial role of the ATN for memory, although the ATN are also considered as important for the pathophysiology of epilepsy and serve as a possible target for DBS treatment in this condition (Aggleton et al., 2010; Fisher et al., 2010). The presence of slow- and fast-spiking bursting anterior thalamic units, which discharge within the theta frequency, suggest that the anterior thalamus is involved in the propagation of theta signals through Papez' pathway (Vertes et al., 2001; Tsanov et al., 2011b). Such theta propagation could have resulting mnemonic functions. The large populations of head direction cells recorded in the anterodorsal and anteroventral thalamic nuclei indicate that the anterior thalamus plays an important role in spatial navigation. Furthermore, the central position of the anterodorsal and anteroventral thalamic nuclei in the hierarchically-organized head direction circuitry (Clark and Taube, 2012) and the apparent integration of theta and head direction information at the level of anteroventral thalamic nucleus (Tsanov et al., 2011a) underline the importance of this region for spatial orientation. Evidence to date suggests that the ATN serve as a subcortical gate for information used in path integration processes by cortical structures. A final point is that, by framing the contributions of the ATN within Papez' circuit, there is the strong implication that the functions of these nuclei are principally driven by the hippocampus. In fact, actions in the opposite direction may prove to be equally crucial. Just as the head direction system relies on inputs from “lower” sites within the tegmentum, i.e., inputs independent of the hippocampus, so there is reason to believe that other tegmental inputs (e.g., from the ventral tegmental nucleus of Gudden and from the lateral dorsal tegmental nucleus) will prove vital in understanding the broader role of these diencephalic nuclei in supporting memory (Vann, 2009). Consequently, these tegmental inputs may also prove to be of considerable importance for medial temporal lobe activity.

### Conflict of interest statement

The authors declare that the research was conducted in the absence of any commercial or financial relationships that could be construed as a potential conflict of interest.
